# Tracing exogenous surfactant in vivo in rabbits by the natural variation of ^13^C

**DOI:** 10.1186/s12931-019-1124-9

**Published:** 2019-07-18

**Authors:** Sonia Giambelluca, Francesca Ricci, Manuela Simonato, Luca Vedovelli, Umberto Traldi, Alessio Correani, Costanza Casiraghi, Matteo Storti, Arianna Mersanne, Paola Cogo, Fabrizio Salomone, Virgilio P. Carnielli

**Affiliations:** 1 Department of Women’s and Children’s Health, University of Padova, Padova, Italy; 2 PCare Laboratory, Fondazione Istituto di Ricerca Pediatrica Città della Speranza, Corso Stati Uniti, 4F, Padova, 35121 Italy; 3 R&D Department, Chiesi Farmaceutici, Parma, Italy; 4 Institute of Anesthesiology and Intensive Care, Department of Medicine – DIMED, University of Padova, Padova, Italy; 5 Thermo Fisher Scientific Spa, Rodano, Milan, Italy; 6 Division of Neonatology, Department of Clinical Sciences, Polytechnic University of Marche and Azienda Ospedaliero-Universitaria Ospedali Riuniti, Ancona, Italy; 7 Department of Medicine, University of Udine, Udine, Italy

**Keywords:** Respiratory distress syndrome, Lung surfactant, Surfactant replacement therapy, ^13^C natural abundance, Stable isotope

## Abstract

**Background:**

Respiratory Distress Syndrome (RDS) is a prematurity-related breathing disorder caused by a quantitative deficiency of pulmonary surfactant. Surfactant replacement therapy is effective for RDS newborns, although treatment failure has been reported. The aim of this study is to trace exogenous surfactant by ^13^C variation and estimate the amount reaching the lungs at different doses of the drug.

**Methods:**

Forty-four surfactant-depleted rabbits were obtained by serial bronchoalveolar lavages (BALs), that were merged into a pool (BAL pool) for each animal. Rabbits were in nasal continuous positive airway pressure and treated with 0, 25, 50, 100 or 200 mg/kg of poractant alfa by InSurE. After 90 min, rabbits were depleted again and a new pool (BAL end experiment) was collected. Disaturated-phosphatidylcholine (DSPC) was measured by gas chromatography. DSPC-Palmitic acid (PA) ^13^C/^12^C was analyzed by isotope ratio mass spectrometry. One-way non-parametric ANOVA and post-hoc Dunn’s multiple comparison were used to assess differences among experimental groups.

**Results:**

Based on DSPC-PA ^13^C/^12^C in BAL pool and BAL end experiment, the estimated amount of exogenous surfactant ranged from 61 to 87% in dose-dependent way (*p* < 0.0001) in animals treated with 25 up to 200 mg/kg. Surfactant administration stimulated endogenous surfactant secretion. The percentage of drug recovered from lungs did not depend on the administered dose and accounted for 31% [24–40] of dose.

**Conclusions:**

We reported a risk-free method to trace exogenous surfactant in vivo. It could be a valuable tool for assessing, alongside the physiological response, the delivery efficiency of surfactant administration techniques.

## Background

RDS is a respiratory disorder that mostly affects preterm infants and it is characterised by lung immaturity and critically low amounts of pulmonary surfactant [1, 2]. To overcome pulmonary surfactant deficiency, exogenous surfactant replacement therapy represents a crucial achievement in the care of the preterm newborn [3], reducing death and pneumothorax [4]. There are several modalities to administer exogenous surfactant, including InSurE (*In*tubation-*Sur*factant-*E*xtubation) [5], aerosolization [6], laryngeal mask [7], and intratracheal catheters [8]. Among these, InSurE procedure has been widely used since it has been shown to improve gas exchange and survival, and to reduce the duration of mechanical ventilation. However, treatment failure after InSurE has been reported to be from 9 to 50% [9], with the need of repeating the treatment in some patients [10].

During clinical development of a therapy, definition of the dosage and dosage schedule is a key question, and it is the objective of the so-called dose-finding studies. [11] Although the performance of a therapy can be evaluated by different clinical parameters, monitoring the fate of a drug in vivo remains a main concern, since it usually entails the need for dispensing labelled compounds [12]. The possibility of estimating the amount of drug reaching the target organ could improve treatment dosing strategies, help in comparing different delivery systems, thus increasing the therapy success rate.

We recently developed a new method to estimate the contribution of exogenous surfactant to the alveolar pool in a rabbit model of RDS based on carbon stable isotope natural abundance of the disaturated-phosphatidylcholine palmitic acid (DSPC-PA) [13], the main phospholipid component of pulmonary surfactant. Briefly, we verified the suitability of our method in lung-lavaged adult rabbits by assessing the constancy of poractant alfa DSPC-PA carbon stable isotopes ratio among different batches, the difference in carbon stable isotopes abundance between endogenous and exogenous surfactant, and the effect of poractant alfa administration on rabbit alveolar DSPC-PA ^13^C content. Our novel method allowed to discriminate an endogenous/exogenous (and vice versa) ratio of 1/49 (2%), without the need for dispensing any artificially labelled compound [14, 15]. This aspect makes the stable isotope natural abundance approach safe and exploitable to study in vivo different doses and surfactant administration methods.

In the present work, we further investigated the efficacy and the reliability of our method in lung-lavaged adult rabbits managed with a nasal continuous positive airway pressure [16] (nCPAP) and treated with increasing doses of poractant alfa in a dose-ranging study.

## Materials and methods

### Animals and sample collection

The experimental procedure was approved by the intramural Animal Welfare Body and the Italian Ministry of Health (Prot. n° 1300–2015-PR) and complied with the European and Italian regulations for animal care. A total of 44 adult rabbits were included in the study. Rabbits (male) were 7 to 8 weeks old, with a body weight of 1.9 ± 0.3 kg (min 1.2, max 2.8 Kg). Water and diet were provided ad libitum. The diet was the same for all animals (3409 Rabbit, maintenance and breeding - Kliba Nafag, Kaiseraugst, Switzerland) and was kept constant for at least six days before the studies.

All rabbits underwent a standardized surfactant depletion procedure, using 20 mL/kg of pre-warmed saline (37 °C) until visual inspection showed transparent lavage fluid, as previously described [16].

For each rabbit, the entire volume of all bronchoalveolar lavages (BALs) necessary for surfactant depletion was pooled (BAL pool). After surfactant depletion, rabbits were randomly assigned to 5 treatment groups and received different doses of poractant alfa using the InSurE technique according to a procedure previously described [16]: 0 mg/kg (*N* = 11); 25 mg/kg (*N* = 6); 50 mg/kg (N = 11); 100 mg/kg (*N* = 8); 200 mg/kg (N = 8).

The dose-range 50–200 mg/kg was previously used to assess the performances of poractant alfa [17] and synthetic surfactant CHF5633 [18].

Ninety minutes after exogenous surfactant administration, all rabbits underwent a second surfactant depletion with the same technique as described above. For each animal, all BALs performed at the end of the experiment were pooled (BAL end experiment).

After homogenization, a 10-mL aliquot of the BAL pool and of BAL end experiment was immediately centrifuged at 100×g for 10 min in order to sediment cells and debris. The supernatant was transferred in a new tube, aliquoted and stored at − 80 °C until analysis.

Aliquots of all the lots of poractant alfa used for the study were provided by Chiesi Farmaceutici (Parma, Italy) and kept at 4 °C until analysis.

### Extraction and isolation of DSPC

BALs were slowly thawed on ice, then homogenised by vortex for 10 s. Lipids were extracted by the method of Bligh and Dyer [19] after addition of the internal standard, dipentadecanoyl phosphatidylcholine (PC-C15) 1 mg/ml. Extracted lipids were oxidized with osmium tetroxide, resuspended in chloroform and spotted on silica gel G thin-layer plates (Merck, Darmstadt, Germany). The plates were developed with chloroform:methanol:isopropanol:0.25% KCl:trimethylamine (40:12:33:8:24).

DSPC was visualized against a standard and scraped off from the silica gel plate. DSPC saturated fatty acids were derivatized as methyl ester by adding 2-mL 3 M HCl methanol and heating at 100 °C for 1 h. Methyl esters were then extracted with hexane.

Poractant alfa aliquots were heated at 37 °C in a water bath, then diluted 1:81 with NaCl 0.9%, processed and analysed in triplicate by following the same procedure used for BAL lipids extraction.

### Stable isotopes analysis

In order to assess DSPC concentration, a prior quantitative analysis of DSPC was performed by Gas Chromatography - Flame Ionization Detector (GC-FID, HP 5890, Agilent Technologies, Santa Clara, CA, USA). DSPC-PA ^13^C/^12^C ratio was analysed by Gas chromatography – Combustion – Isotope Ratio Mass Spectrometry (GC-C-IRMS, Delta V Advantage, Thermo Fisher Scientific, Waltham, MA, USA).

For GC-C-IRMS analysis, the DSPC fatty acids separation was achieved on an Ultra-2 (25 m, 0.32 mm, 0.52-μm film thickness) column (Agilent). Helium was used as carrier gas, and on-column injection was applied. The initial temperature gradient was 60 °C, 1-min hold; then increased to 195 °C with a rate of 30 °C/minute, 2-min hold; finally, 30 °C/min to 240 °C, with a hold of 7 min.

Fatty acids were then combusted online at 960 °C and introduced as CO_2_ into the ion source. Finally, the ^13^C/^12^C ratio of the ionized CO_2_ was measured by detecting mass 44/45.

Carbon isotopic analysis was performed in triplicate and normalized against Vienna Pee Dee Belemnite (V-PDB) standard. Results are expressed as δ‰ (delta per-mil) of ^13^C/^12^C, which indicate the difference, in part per thousand, from the standard:

$$ \delta X\ \left({\mbox{\fontencoding{U}\fontfamily{wasy}\selectfont\char104}} \right)=\frac{R\  sample-R\  standard}{R\  standard}\ast 1000 $$

where “R” is the ratio of the heavy to light isotope in the sample or standard.

### Calculations

Data analysis was carried out with Microsoft Excel 2016 (Microsoft Corp). The contribution of exogenous surfactant to the alveolar pool was calculated as follows:

$$ \mathrm{BAL}\ \mathrm{DSPC}\left(\mathrm{mg}/\mathrm{Kg}\right)=\frac{\mathrm{DSPC}\ \left(\mathrm{mg}/\mathrm{ml}\right)\ast \mathrm{BAL}\ \mathrm{Volume}\ \left(\mathrm{ml}\right)}{\mathrm{Rabbit}\ \mathrm{body}\ \mathrm{weight}\ \left(\mathrm{Kg}\right)} $$

$$ \mathrm{Dilution}\ \mathrm{factor}=\frac{\mathrm{PA}\ 13\mathrm{C}/12\mathrm{C}\ \mathrm{of}\ \mathrm{BAL}\ \mathrm{end}\ \mathrm{experiment}-\mathrm{PA}\ 13\mathrm{C}/12\mathrm{C}\ \mathrm{of}\ \mathrm{BAL}\ \mathrm{pool}}{\mathrm{PA}\ 13\mathrm{C}/12\mathrm{C}\ \mathrm{of}\ \mathrm{administered}\ \mathrm{DSPC}-\mathrm{PA}\ 13\mathrm{C}/12\mathrm{C}\ \mathrm{of}\ \mathrm{BAL}\ \mathrm{pool}} $$

$$ \mathrm{Exogenous}\ \mathrm{DSPC}\ \left(\mathrm{mg}/\mathrm{Kg}\right)=\mathrm{DSPC}\ \mathrm{in}\ \mathrm{BAL}\ \mathrm{end}\ \mathrm{experiment}\ \left(\mathrm{mg}/\mathrm{Kg}\right)\ast \mathrm{dilution}\ \mathrm{factor} $$

$$ \mathrm{Endogenous}\ \mathrm{DSPC}\ \left(\mathrm{mg}/\mathrm{Kg}\right)=\mathrm{Total}\ \mathrm{DSPC}\ \mathrm{in}\ \mathrm{BAL}\ \mathrm{end}\ \mathrm{experiment}-\mathrm{exogenous}\ \mathrm{DSPC} $$

$$ \%\mathrm{of}\ \mathrm{administered}\ \mathrm{in}\ \mathrm{lungs}=\frac{\mathrm{administered}\ \mathrm{DSPC}\ \mathrm{x}\ 100}{\mathrm{Exogenous}\ \mathrm{DSPC}\ } $$

Data for each lot of administered poractant alfa were used for the calculations relative to the receiving rabbits.

For rabbits receiving 0 mg/kg of poractant alfa the DSPC recovered in BAL end experiment was assumed to be 100% endogenous.

### Statistical analysis

Wilcoxon signed rank test was performed to compare carbon stable isotopes ratio in BAL pool and BAL end experiment of control rabbits. One-way non-parametric ANOVA (Kruskal-Wallis) and post-hoc Dunn’s multiple comparison were used to assess differences among experimental groups.

Kendall’s Tau-b (τb) coefficient of correlation was used to measure the degree of association between the dose administered and changes in DSPC amount and carbon stable isotopes content in treated rabbits.

Statistical analysis was performed using GraphPad Prism 5 (GraphPad Software Inc., San Diego, CA, USA) and SPSS 25 (IBM Corp, Armonk, NY, USA) software. A *p*-value < 0.05 was considered significant. Data are presented as median and interquartile range [IQR] or as mean ± SD, according to data distribution.

## Results

**Fig. 1:**
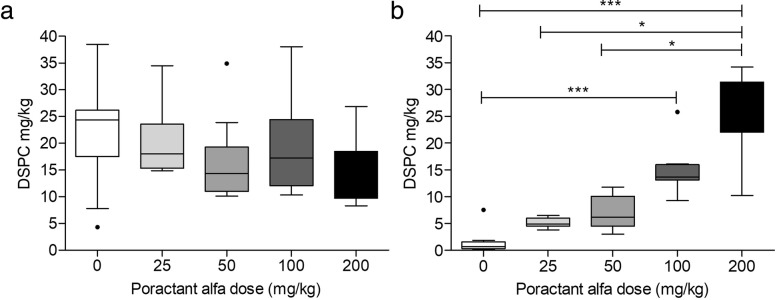
Alveolar DSPC amount (mg/kg) in BALs of rabbits treated with different doses of poractant alfa. (**a**) BAL pool: pool of all depletion BALs; (**b**) BAL end experiment: pool of all BALs collected after poractant alfa administration. Data shown as median and interquartile range. Statistical differences according to Dunn’s multiple comparison: *p < 0.05, ***p < 0.001. Kendall’s correlation for Fig. 1b: τb = 0.789, *p* < 0001

BAL pool and BAL end experiment from 44 rabbits treated with different doses of poractant alfa were analysed for DPSC content and DSPC-PA ^13^C/^12^C ratio to assess the contribution of exogenous surfactant to the alveolar pool. The alveolar DSPC content in BAL pool and BAL end experiment of rabbit experimental groups are shown in Fig. 1. No differences were found for BAL pool (*p* = 0.177), while the DSPC amount in BAL end experiment was significantly different (*p* < 0.0001) and positively correlated (τb 0.789, *p* < 0001) with the dose administered.

The ^13^C abundance of alveolar DSPC-PA was constant during the experiment for rabbits treated with 0 mg/kg of poractant alfa: BAL pool − 29.1‰ [− 29.5, − 28.8]; BAL end experiment − 29.0‰ [− 29.3, − 28.1] (*p* = 0.41). A positive correlation (τb 0.654, *p* < 0.0001) was found between the differences in ^13^C/^12^C of DSPC-PA in BAL end experiment and BAL pool and the administered dose.

**Fig. 2:**
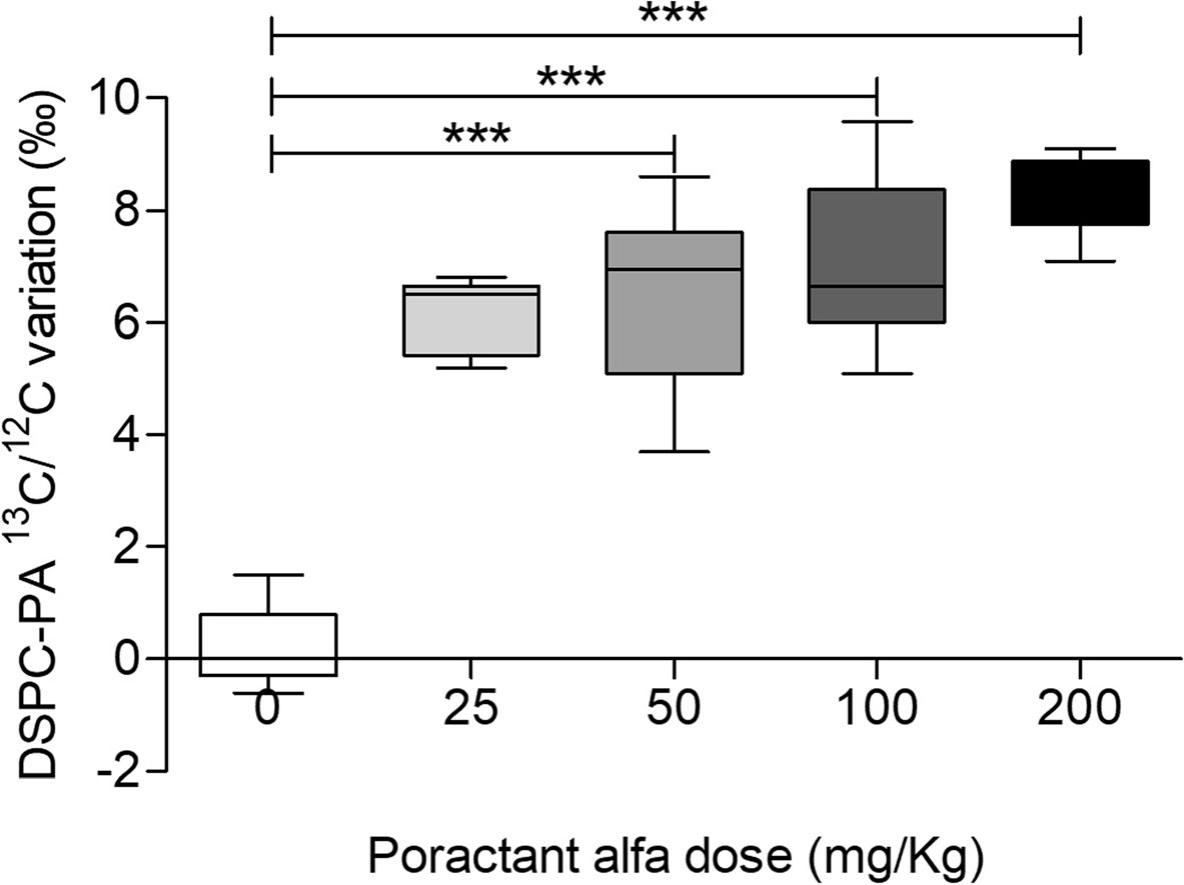
Variation of DSPC-PA ^13^C. Differences between alveolar DSPC-PA^13^C/^12^C in BAL end experiment and BAL pool of rabbits treated with different doses of poractant alfa. BAL pool: pool of all depletion BALs; BAL end experiment: pool of all BALs collected after poractant alfa administration. Data shown as median and interquartile range. Statistical differences according to Dunn’s multiple comparison: ***p < 0.001. Kendall’s correlation: τb = 0.654, *p* < 0001

Comparison of the difference between DSPC-PA ^13^C/^12^C in BAL end experiment and BAL pool in rabbits treated with different doses of poractant alfa is shown in Fig. 2. All groups (50 mg/kg 6.9‰ [5.1, 7.6]; 100 mg/kg 6.7‰ [6.0, 8.4]; 200 mg/kg 8.2‰ [7.8, 8.9]), except animals treated with a dose of 25 mg/kg (6.5‰ [5.4, 6.7]), significantly differed from rabbits treated with 0 mg/kg (0.0‰ [− 0.3, 0.8]) (*p* < 0.0001).

Based on the differences on ^13^C/^12^C of alveolar DSPC-PA in BAL pool and BAL end experiment, the contribution of exogenous DSPC to the rabbit alveolar pool and the percentage of administered poractant alfa recovered from the lungs were calculated in all treated rabbits. The percentage of administered poractant alfa recovered from the lungs did not correlate with the administered dose (*p* = 0.80) and accounted for 38% [20, 43], 24% [14, 48], 29% [21, 22] and 35% [23, 40] of the dose with 25, 50, 100, and 200 mg/kg, respectively.

**Fig. 3:**
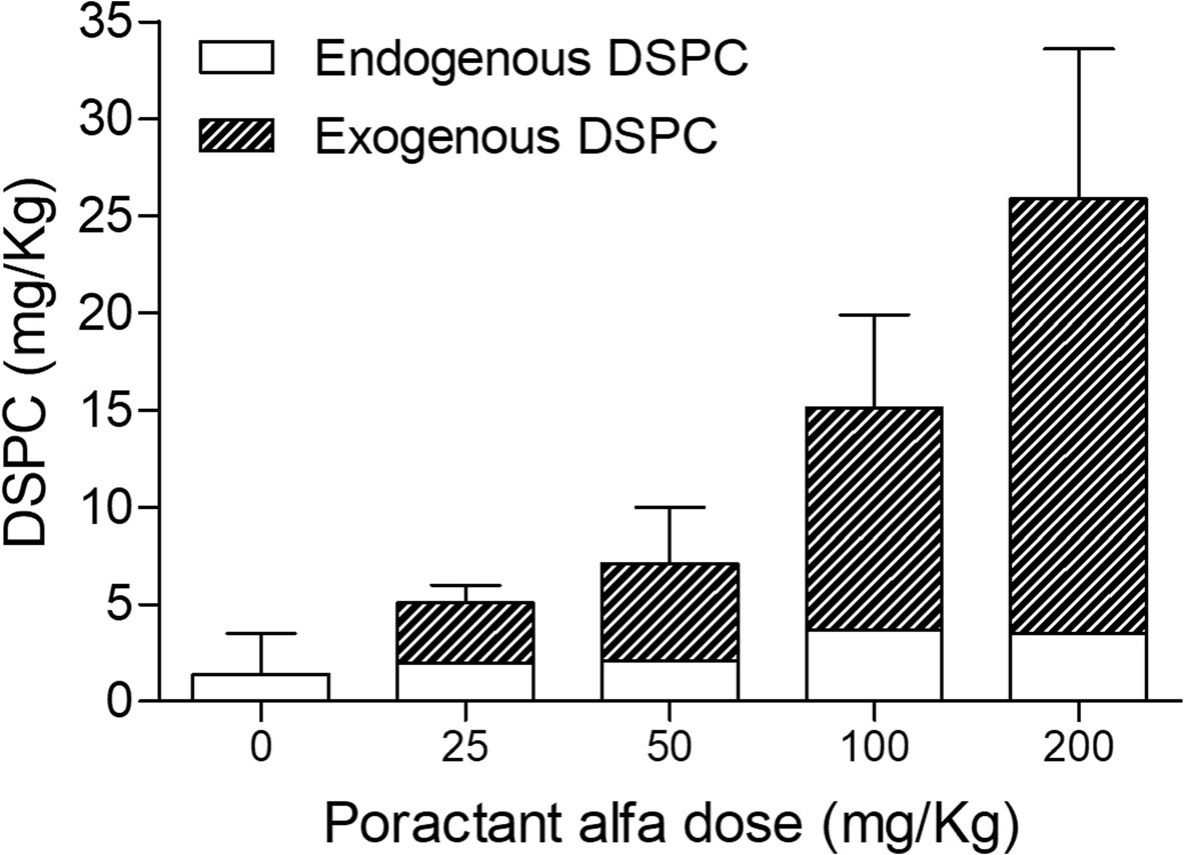
Estimated contribution of exogenous surfactant. Ratio between exogenous and endogenous DSPC (mg/kg) in the alveolar pool of rabbits treated with different doses of poractant alfa by InSurE. Data shown as mean. Standard deviation bars refer to the total amount of DSPC. Kendall’s correlation for the exogenous surfactant dose: τb = 0.710, *p* < 0.0001

The mean ratio between endogenous and exogenous DSPC in the rabbit alveolar pool of the different experimental groups is shown in Fig. 3. The estimated amount of exogenous surfactant in the alveolar pool increased in a dose-dependent way (τb 0.710, *p* < 0.0001). A significantly higher amount was found for 200 mg/kg group (24.4 mg/kg [19.1, 26.7]) compared to rabbits treated with 25 mg/kg (3.0 mg/kg [2.5, 3.7], *p* < 0.001) and 50 mg/kg (4.1 mg/kg [2.7, 8.5], p < 0.001), and for 100 mg/kg group (9.4 mg/kg [8.9, 12.1]) compared to rabbits treated with 25 mg/kg (*p* < 0.05).

The estimated amount of endogenous surfactant secreted into the airways during 90 min after exogenous surfactant administration was higher in rabbits treated with 100 mg/kg (4.0 mg/kg [2.2, 4.3]) and 200 mg/kg (3.8 mg/kg [2.7, 4.6]) than in rabbits receiving 0 mg/kg of poractant alfa (0.7 mg/kg [0.3, 1.6]) (*p* < 0.01).

## Discussion

In the present study, we showed the feasibility of using the ^13^C natural abundance approach to trace exogenous surfactant (poractant alfa) at progressively increasing doses in a rabbit model of RDS. To this purpose, we verified: the constancy of alveolar DSPC-PA ^13^C/^12^C during the experiment in a group of untreated rabbits (0 mg/kg dose); the correlation between the dose administered and changes in alveolar DSPC-PA ^13^C/^12^C before and after treatment with poractant alfa. Since these premises were satisfied in our model, we calculated the contribution of poractant alfa to the surfactant alveolar pool. Dose-dependent studies have been previously published for different animal-derived surfactants, including bovactant (Alveofact, Boehringer-Ingelheim, Ingelheim am Rhein, Germany) [20] and poractant alfa [17, 18]. However, those studies focused on the clinical effects of the administered dose and on the performance of the exogenous surfactant. Data about the amount of drug reaching the lungs are scarce. Here we investigated the possibility to estimate the percentage of surfactant delivered to the lung by InSurE procedure at increasing doses of the drug. The percentage of administered exogenous surfactant recovered from the BAL was only 31%. This is because an important fraction of the administered surfactant becomes inaccessible to lavage already at 1 min after administration [21]. Moreover, the percentage of recovery was independent from the dose instilled. This is a crucial result for allowing users to confidently apply this technique in different surfactant replacement studies where often different surfactant dosages need to be compared. There was a positive correlation between the administered dose and the differences in DSPC-PA ^13^C/^12^C before and after treatment. Based on the multiple comparison test used, significant difference in the variation of ^13^C was not reached only for the comparison between rabbits receiving 0 and 25 mg/kg of surfactant, because the reported *p*-value is adjusted for the number of rabbits (11 vs 6, respectively). These data, taken together, corroborate the power of our method, highlighting both a standardization of drug administration and BAL collection procedures on one side and the sensitiveness of the natural abundance approach to the dose administered on the other. Of interest, we used doses of poractant alfa which are much lower than the recommended dose of 200 mg/kg. We showed the suitability of our method to quantify “rather low” therapeutic doses allowing the premises to apply this approach also for animal studies on new less-invasive surfactant application (LISA) systems or for aerosolized or nebulized surfactant administrations, exploiting the present results as a calibration curve for the total delivered surfactant dose.

The combination of both quantitative data on DSPC amount and on carbon modification after treatment resulted in a clear picture of DSPC alveolar pool, which was a combination of both poractant alfa and new synthetized and/or secreted DSPC. The possibility to accurately estimate the ratio between the exogenous and the alveolar DSPC also allowed for the knowledge that a greater endogenous portion can be detected in rabbits who underwent therapy (at clinical dosages) compared to animals who did not receive the drug. The effect of the administration of exogenous surfactant on the synthesis and secretion of endogenous lung surfactant have been extensively studied, although the results are still controversial [22–24]. In vivo studies suggested an enhanced synthesis of endogenous surfactant PC both in rabbits [25, 26] and preterm infants [27], while no noticeable effects on the endogenous synthesis have been described in mice treated with a therapeutic dose of both synthetic or natural exogenous surfactant [28]. In the present study, a clear dose-dependent tendency was observed for the comparison of the endogenous DSPC amount in the alveolar pool among the experimental groups, confirming a stimulation of endogenous DSPC secretion or synthesis after treatment, mainly when a therapeutic dose is administered. Of note, the present study was conducted in adult rabbits thus with mature lungs and, likely, with normal amounts of intracellular surfactant. Preterm infants with RDS have low amounts of both alveolar and lung tissue associated surfactant and in addition DSPC synthesis is much reduced. We speculate that endogenous surfactant synthesis/secretion is less likely to occur in preterm infants in the time frame of our animal studies. Pilot feasibility studies in preterm infants are ethically acceptable and they need to be done. Worth of note, in previous reports the contribution of newly synthesized surfactant has been estimated by the use of radio-labelled [24, 29] or stable isotope-labelled precursors of surfactant phospholipids [27, 28]. This is the first study in which the exogenous and endogenous surfactant can be distinguished in the alveolar pool without the need for administering any artificially labelled compound. This aspect supports a higher suitability of our method for research in human medicine, even for in vivo studies involving a vulnerable target population like preterm infants.

This approach is non-invasive and risk-free, but studies in infants carry some limitations. The first is that it requires patients to be intubated to allow for airways sampling. Sample collection is not possible in case of non-invasive surfactant administration techniques, however the need for reintubation after such techniques was reported to be as high as 75% [30]. Data on the contribution of exogenous surfactant to the total amount of surfactant from tracheal aspirates in case of reintubation after the first surfactant administration is of interest to clinicians and it may help in understanding the cause of the respiratory failure. Our method could help in distinguishing respiratory (surfactant related) from non-respiratory causes of CPAP failure, and could provide information on the endogenous or exogenous origin of the surfactant deficiency [31]. Our technique has theoretical limitations as well. One of these could be represented by the inter-individual variability of ^13^C content. This approach is based on a different isotopic background between the drug and the biological pool. It is well known that in animal tissues ^13^C values typically reflect those of diet [32]. Here we reported a study conducted on rabbits with a controlled diet. A restriction could be represented by the inconsistency of carbon stable isotope background moving from the animal model to infants fed with variable diets. We have no data on the feasibility of our method in countries where the ^13^C dietary abundance of the population is high as this will likely be the case of countries with high corn-based food consumption [33, 34]. Data collection about intra- and inter-individual variability of DSPC-PA ^13^C/^12^C in infants who needed intubation are under way.

Despite these limitations, if this method will be proven to be suitable for clinical applications in human research, this could represent a significant advancement in surfactant replacement therapy for the treatment of RDS, especially in extremely low birth weight infants.

Under the premises of an appreciable difference in stable isotope content between the exogenous compound and the biological counterpart, and of a constancy of drug stable isotope content in different lots, this method could also be extended to other drug therapies.

## Conclusion

We demonstrated the reliability of carbon stable isotopes approach at natural abundance to trace in vivo the fate of surfactant replacement therapy. Since the administered drug behaves as a natural tracer, no other labelled compound is required. This approach could be a valuable tool for assessing, alongside the physiological response, the delivery efficiency of different less-invasive administration techniques in in vivo studies. However, the feasibility of this approach in infants deserves further investigations.

## Data Availability

The datasets used and/or analysed during the current study are available from the corresponding author on reasonable request.

## Abbreviations

BAL: Bronchoalveolar Lavage
DSPC: Disaturated-phosphatidylcholine
GC-C-IRMS: Gas Chromatography – Combustion – Isotope Ratio Mass Spectrometry
GC-FID: Gas Chromatography - Flame Ionization Detector
InSurE: Instillation Surfactant Extubation
IQR: Interquartile Range
LISA: Less Invasive Surfactant Administration
nCPAP: nasal Continuous Positive Airway Pressure
PA: Palmitic Acid
PC: Phosphatidylcholine
RDS: Respiratory Distress Syndrome
V-PDB: Vienna Pee Dee Belemnite
